# Sod2 haploinsufficiency does not accelerate aging of telomere dysfunctional mice

**DOI:** 10.18632/aging.100030

**Published:** 2009-03-05

**Authors:** Luis Miguel Guachalla, Zhenyu Ju, Rafal Koziel, Guido von Figura, Zhangfa Song, Markus Fusser, Bernd Epe, Pidder Jansen-Dűrr, K. Lenhard Rudolph

**Affiliations:** ^1^Institute of Molecular Medicine and Max-Planck-Research-Group on Stem Cell Aging, University of Ulm, 89081 Ulm, Germany; ^2^International M.D./Ph.D. Program, Medical School Hannover, Germany; ^3^Institute for Biomedical Aging Research, Austrian Academy of Sciences, A-6020 Innsbruck, Austria; ^4^Department of Internal Medicine I, University of Ulm, Germany; ^5^Institute of Laboratory Animal Sciences and Max-Planck-Partner Group on Stem Cell Aging, Chinese Academy of Medical Sciences, Beijing, China; ^6^Institute of Pharmacy, University of Mainz, D-55099 Mainz, Germany

**Keywords:** oxidative stress, superoxide, telomere shortening, aging, DNA damage, SOD2, free radicals, stem cells

## Abstract

Telomere shortening represents a causal factor of cellular senescence. At the same time, several lines of evidence indicate a pivotal role of oxidative DNA damage for the aging process *in vivo*. A causal connection between the two observations was suggested by experiments showing accelerated telomere shorting under conditions of oxidative stress in cultured cells, but has never been studied *in vivo*. We therefore have analysed whether an increase in mitochondrial derived oxidative stress in response to heterozygous deletion of superoxide dismutase (*Sod2*^+/-^) would exacerbate aging phenotypes in telomere dysfunctional (*mTerc*^-/-^) mice. Heterozygous deletion of *Sod2* resulted in reduced SOD2 protein levels and increased oxidative stress in aging telomere dysfunctional mice, but this did not lead to an increase in basal levels of oxidative nuclear DNA damage, an accumulation of nuclear DNA breaks, or an increased rate of telomere shortening in the mice. Moreover, heterozygous deletion of *Sod2* did not accelerate the depletion of stem cells and the impairment in organ maintenance in aging *mTerc*^-/-^ mice. In agreement with these observations, *Sod2* haploinsufficiency did not lead to a further reduction in lifespan of *mTerc^-/-^* mice. Together, these results indicate that a decrease in SOD2-dependent antioxidant defence does not exacerbate aging in the context of telomere dysfunction.

## Introduction

The free radical theory of aging proposes that free radicals accelerate the accumulation of damaged structures over time leading to impaired cellular and organismal function during aging [1]. Oxidative stress is driven by reactive oxygen species mainly produced in mitochondria. Superoxide anions, being produced at complex I and III of the electron transport chain [2], are primarily detoxified in mitochondria by the manganese dependent form of superoxide dismutase SOD2 (also called MnSOD). It has been shown that *Sod2* over-activation can prolong the lifespan of yeast [3, 4]. *Vice versa*, impairment or deletion of SOD2 expression induced a significant shortening of the lifespan of *Drosophila* [5, 6] and mice [7, 8].

Mice carrying a heterozygous deletion of *Sod2* (*Sod2^+/-^*) are viable but show increased oxidative stress, increased nuclear and mitochondrial DNA modifications, impaired mitochondria function, and increased apoptosis rates [9-13]. *Sod2^+/-^* mice exhibit slightly increased rates of cancer but no other features of accelerated aging and have a normal lifespan [13]. These studies indicated that a decrease in the oxidative damage defense system by itself does not induce a significant increase in aging pathology in mice. However, the *Sod2^+/-^*mouse provided a unique experimental system to analyze whether an impaired anti-oxidant defense can cooperate with other molecular causes of aging and disease. Although *Sod2* polymorphisms were not associated with longevity of centenarians [14], the investigation of this question appears to be highly relevant since SNPs in various components of the oxidative stress pathway are associated with phenotypes of human aging [15].

There is growing evidence that an accumulation of telomere dysfunction and DNA damage contributes to human aging  [16, 17]. Several lines of evidence indicate that reduced SOD2 levels and increased ROS could influence cellular and organismal aging in the context of telomere shortening and DNA damage accumulation: (i) Experimental data have shown that ROS can induce different lesions in nuclear DNA including oxidized bases, strand breaks and mutations [18-21]. Thus, ROS may contribute to the generation of nuclear DNA damage and the evolution of aging pathology at organismal level.

(ii) Telomere shortening limits the proliferative capacity of human cells to 50-70 cell divisions by induction of senescence or apoptosis [22-25]. Replicative senescent cells show increased ROS levels [26, 27] and increased oxidative DNA damage [28] indicating that senescence can accelerate ROS induced DNA damage. In addition, increased ROS levels and oxidative modifications to DNA have also been implicated as causal factors inducing senescence [19, 27, 29-33]. In agreement with this hypothesis, it was shown that increased ROS accelerate the rate of telomere shortening in cell culture [34] and that oxidative stress severely limits the replicative potential of mouse cells independently of the presence of telomerase [35]. It remains yet to be investigated whether ROS and telomere shortening cooperate to induce an accumulation of DNA damage and senescence in aging tissues.

(iii) SOD2 level could influence the induction of checkpoints in response to DNA damage or telomere dysfunction. It has been shown that decreased SOD2 expression accelerated p53-induced apoptosis [36], whereas up-regulation of SOD2 protected cells from apoptosis by stabilization of mitochondrial membranes [37]. Both mechanisms could be relevant to aging induced by telomere dysfunction, since the impairment of organ maintenance in response to telomere dysfunction is associated with activation of the p53/p21 signaling pathway and increased rates of apoptosis [38-41].

Laboratory mouse strains are of limited use to identify factors that accelerate aging in the context of telomere dysfunction and DNA damage, since laboratory mice in comparison to humans, have very long telomeres [42]. Laboratory mice show some evidence for an accumulation of DNA damage during aging [43] However, biomarker studies revealed that the level of telomere dysfunction and DNA damage in aging laboratory mice is low compared to human aging [16]. Telomerase knockout (*mTerc^-/-^*) mice provided an experimental system to study aging induced by telomere dysfunction and DNA damage [16, 44, 45]. Considering that oxidative stress was shown to shorten telomeres [46], limit stem cell function [47], and induce DNA damage and senescence (see above), we hypothesized that *Sod2* haploinsufficiency could affect stem cell pools and aging of telomere dysfunctional mice.

Here we analyzed consequences of a heterozygous deletion of *Sod2* on aging of telomerase wild-type mice with long telomeres and third generation (G3) *mTerc^-/-^* mice with dysfunctional telomeres. The study shows that heterozygous *Sod2* deletion does not affect stem cell function, organ maintenance and lifespan of telomere dysfunctional mice. These results indicate that a reduction in SOD2-dependent anti-oxidant defense does not accelerate aging in the context of telomere dysfunction.

## Results

### Heterozygous deletion of* Sod2* reduces SOD2 protein levels and antioxidant capacity

*Sod2^+/-^* mice were crossed through 3 generations with telomerase knockout mice (Suppl. Figure 1) to generate the following cohorts: *mTerc^+^*,*Sod2^+/-^* mice (n=31); *mTerc^+^*, *Sod2^+/+^* mice (n=34); G3 *mTerc^-/-^*, *Sod2^+/-^* mice (n=58); G3 *mTerc^-/-^*, *Sod2^+/+^*mice (n=38). The *mTerc^+^* groups were composed of both *mTerc^+/+^* and *mTerc^+/-^* mice with long telomeres since they do not phenotypically differ from each other.

**Figure 1.**
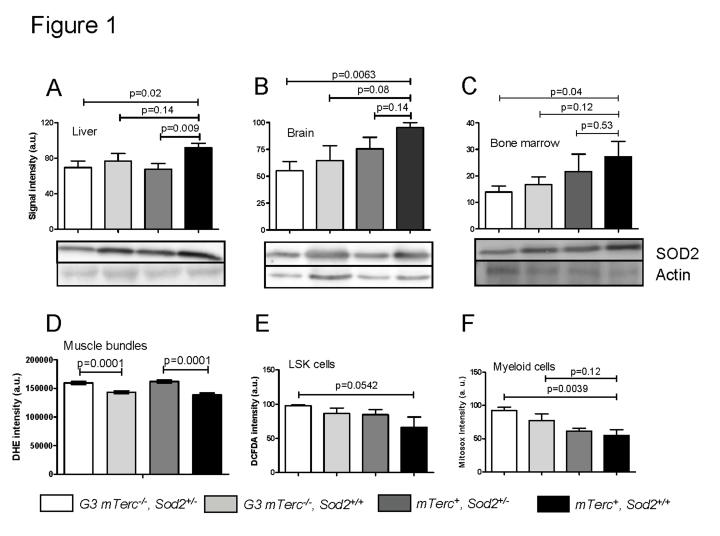
Western blots showing SOD2 levels in liver (**A**), brain (**B**) and bone marrow (**C**) of 12 to 18 months old mice. Lower panels show representative western blots and upper panels show quantification of normalized SOD2 levels to actin controls from n=4 mice per group (1 to 2 repeat experiments per sample). Data is shown in arbitrary units ± SEM. (**D**) Basal ROS levels in muscle fibers stained with DHE. Signal quantification of G3 *mTerc^-/-^Sod2^+/-^* (n=235), G3* mTerc^-/-^*(n=211) *mTerc*^+^*, Sod2^+/-^*(n=270* ) *and *mTerc*^+^*, Sod2^+/+^* (n=203) nuclei from 5 mice per genotype. Data is shown as mean fluorescence intensity ± SEM. (**E**) Antioxidant capacity of LSK cells. DCFDA loaded bone marrow cells were incubated with 50 uM of antimycinA and DCFDA fluorescence was monitored in Lin^-^Sca^+^cKit^+ ^populations by FACS analysis. Data is shown in arbitrary units ± SEM of n=4 mice per group. (**F**) Antioxidant capacity of myeloid cells. Mitosox loaded bone marrow cells  were incubated  with 20 uM  antimycinA and mitosox intensity monitored in myeloid population by FACS analysis.  "Y" axis denotes arbitrary units for fluorescence intensity of n=5 to 6 mice per group.

In *mTerc^+^* mice, heterozygous deletion of *Sod2* correlated with significantly decreased SOD2 protein levels in liver, whereas the decrease in brain and bone marrow did not reach statistical significance (Figure 1A-C). SOD2 protein levels were slightly but not significantly decreased in G3 *mTerc^-/-^*,*Sod2^+/+^* mice compared to *mTerc^+^*, *Sod2^+/+^* mice. A further decrease occurred in G3 *mTerc^-/-^*, *Sod2^+/-^* mice resulting in a significant decrease in SOD2 protein levels in all investigated organs of these mice compared to  *mTerc^+^*, *Sod2^+/+^* mice (Figure 1A-C).

To investigate functional consequences of heterozygous *Sod2* deletion, levels of reactive oxygen species (ROS) were analyzed in muscle and hematopoietic cells. For this purpose we used (i) dihydroethidium (DHE), which intercalates in DNA and emits red fluorescent signals in response to oxidation;  (ii) mitosox,  which localizes  to mitochondria and exhibits red fluorescence after superoxide-induced oxidation, and (iii) dichloro-dihydro-fluorescein (DCFDA), which detects a wide range of ROS after removal of its acetate group by oxidation. Heterozygous deletion of Sod2 was associated with increased basal superoxide levels in muscle cells of both*mTerc^+^*and G3 *mTerc^-/-^* mice (Figure 1D). *Sod2* gene status had no significant effect on basal ROS levels in bone marrow derived stem and progenitorcells (LSK cells: Lineage-negative, Sca1-positive, c-Kit-positive, data not shown). However, stress induced ROS level after treatment with antimycinA (a complex III inhibitor that induces superoxide production) were elevated in bone marrow derived LSK and myeloid cells of G3 *mTerc^-/-^*, *Sod2^+/-^* mice compared to *mTerc^+^*, *Sod2^+/+^* mice (Figure 1E, F). In accordance with the data on SOD2 protein expression, these data on antimycinA induced ROS in hematopoietic and myeloid cells suggested that *Sod2* haploinsufficiency cooperated with telomere dysfunction to impair ROS detoxification in G3 *mTerc^-/-^, Sod2^+/-^* mice.

### *Sod2* heterozygous deletion does not increase mitochondrial dysfunction in aging G*3 mTerc*^-/-^ mice

**Figure 2.**
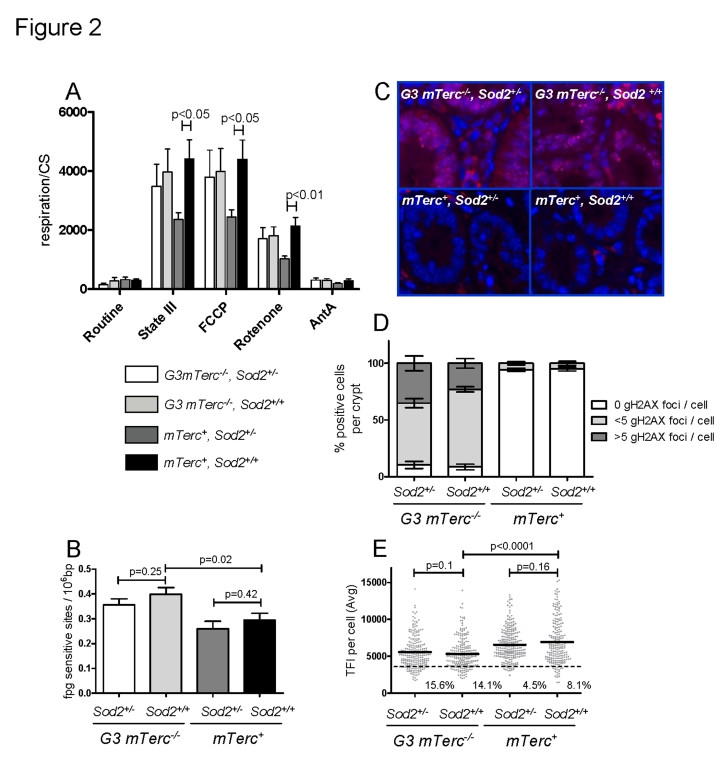
(**A**) Mitochondrial respiration of muscle fibers. 10 to 25 mg of  permeabilized bundles were analyzed by high resolution respirometry. Results are expressed as oxygen consumption per mg of muscle (± SEM) normalized to citrate synthase activity of  n=5 to 6 mice per group. State III respiration is shown after addition of malate, octanoyl-carnitine, ADP, glutamate, succinate and cytochrome c. After state III respiration determination, uncoupled respiration was determined with addition of FCCP to the respiring fibers. Rotenone and antimycin A were used to inhibit respiration at complex I and III respectively. (**B**) Oxidative modifications (fpg sites) in DNA from bone marrow cells of 12 to 17 month old mice. Data from n=4 to 9 mice per group is shown as number of lesions per 10^6^ bp ± SEM. (**C**) Representative pictures of  gH2AX staining in intestinal crypts of aged mice and bar graphs (**D**) showing  percentage of positive cells per crypt and number of foci per cell ± SEM of n=4 to 6 mice per group. 200 crypt cells were analyzed per mouse. (**E**) Telomere length analysis by qFISH in liver sections of n= 4 to 5 mice per group aged 12 to 18 months old. n=237 G3 *mTerc^-/-^, Sod2^+/-^*), n=234 (G3* mTerc^-/-^*, *Sod2^+/+^*); n=242   (*mTerc*^+^*, Sod2^+/-^*) and n=211 (*mTerc*^+^*, Sod2^+/+^*) nuclei were analyzed for telomere fluorescence intensity (TFI). The black line indicates the mean TFI value of each genotype and the dotted line  the threshold of critically short telomeres (TFI<3500).

Mitochondria are the major source of ROS production in cells and a decrease in anti-oxidant defense can induce mitochondrial damage leading to a decrease in the mitochondrial respiratory capacity [9, 48]. In agreement with these studies, muscles from 8-11 month old *mTerc*^+^,*Sod2^+/-^* mice had lower state III respiration rates (ADP dependent, normalized to the mitochondrial mass) and lower maximum respiration rates (induced with the mitochondrial uncoupler FCCP) compared to fibers from *mTerc*^+^,*Sod2^+/+^* mice (Figure 2A). However, this decrease was ameliorated in muscle fibers of 8-11 month old G3* mTerc^-/-^*, *Sod2^+/-^* mice (Figure 2A). Treatment with rotenone, a complex I inhibitor, reduced but did not completely abolish respiration rates of the muscles fibers, indicating ongoing complex I independent respiration. This complex I independent respiration was also significantly reduced in *Sod2^+/-^* mice compared to wild type controls (Figure 2A). Again, this *Sod2*-dependent reduction in respiration rate was rescued in G3* mTerc^-/-^*, *Sod2^+/-^* mice.

An analysis of basal respiration rates and maximal induced respiration (in response to FCCP treatment) did not reveal a significant influence of *Sod2* gene status on the respiratory capacity of total bone marrow cells of 8 to 11 month old *mTerc^+^* and G3 *mTerc^-/-^*mice (Suppl. Figure 2A). In this compartment, mitochondria from G3 *mTerc^-/-^* mice showed an increased maximal respiratory capacity (FCCP-induced) compared to *mTerc^+^* mice suggesting that telomere dysfunction induced adaptive responses that increase the functional reserve of mitochondria.

### *Sod2* heterozygous deletion does not increase nuclear DNA damage and telomere shortening in aging G*3mTerc*^-/-^ mice

To analyze the basal levels of oxidative purine modifications in DNA from total bone marrow cells, we used an alkaline elution assay in combination with formamidopyrimidine-DNA glycoslyase (Fpg) as a probe [49, 50]. The enzyme recognizes 7,8-dihydro-8-oxoguanine (8-oxodG) among other oxidative purine lesions in nuclear DNA [51]. The technique avoids the spontaneous generation of 8-oxodG during DNA isolation and hydrolysis [52]. Our analysis revealed an increase of the basal level of oxidative damage in the nuclear DNA of G3 *mTerc^-/-^* mice compared to *mTerc^+^* mice, but heterozygous deletion of *Sod2* had no influence (Figure 2B).

Increased ROS levels have been shown to induce DNA double strand breaks and senescence in response to prolonged interferon stimulation [19]. Here, the prevalence of DNA double strand breaks was analyzed by γH2AX staining. γH2AX forms foci at DNA breaks in  response to  telomere dysfunction  and  γ-irradiation [53-56]. DNA damage foci were analyzed in intestinal crypts (a proliferative stem cell compartment, which is highly sensitive to telomere dysfunction). In agreement with previous studies, 12-18 month old G3 *mTerc-/-* mice exhibited significantly higher levels of DNA damage compared to age-matched *mTerc+* mice (Figure 2C-D). However, heterozygous deletion of *Sod2* did not increase accumulation of DNA damage foci (Figure 2C-D).

Increasing ROS levels can accelerate telomere shortening in cell culture systems [34, 46]. As expected, an analysis of telomere length showed shorter telomeres in liver (Figure 2E) and intestine (Suppl. Figure 2B) of 12 -18 month old G3  *mTerc^-/-^* mice compared to age-matched *mTerc^+^* mice. However, *Sod2* haplo-insufficiency did not accelerate telomere shortening (Figure 2E and Suppl. Figure 2B). In agreement with the data on oxidative DNA damage, γH2AX-foci, and telomere length, the expression level of serum markers of DNA damage and telomere dysfunction [16] was increased in 12 to 18 month old G3 *mTerc^-/-^* mice compared to age-matched *mTerc^+^* mice, but *Sod2* haploinsufficiency did not increase serum levels of these biomarkers (Suppl. Figure 2C-E).

### *Sod2* heterozygosity does not aggravate the impairment of stem cell function, organ maintenance, and the shortening in lifespan of telomere dysfunc-tional mice

**Figure 3.**
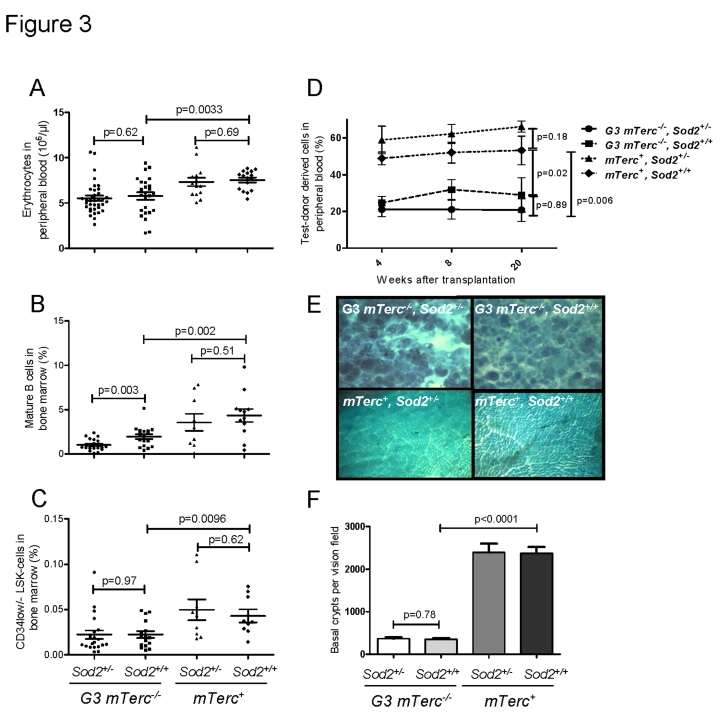
(**A**) Number of erythrocytes per ul of peripheral blood ± SEM in 12 to 18 months old mice. (**B**) Percentage of mature B cells defined as IgD^+^ IgM^+^ B220^+^ CD43^- ^cells in total bone marrow cells of 12 to 18 months old mice. n=21 (G3 *mTerc^-/-^, Sod2^+/-^*), n=17 (G3* mTerc^-/-^*, *Sod2^+/+^*); n=9   (*mTerc*^+^*, Sod2^+/-^*) and n=12 (*mTerc*^+^*, Sod2^+/+^*) mice per group were analyzed by FACS. (**C**) Percentage of long term hematopoietic stem cells defined as Lin^-^ Sca^+^ cKit^+^ CD34^-/low^ cells in total bone marrow cells of 12 to 18 months old mice. n=9 to 20 mice per group were analyzed by FACS. (**D**) Competitive transplantation of total bone marrow of Ly5.2 test donor cells against Ly5.1 competitor cells. 8(10)^5^ cells of test donor cells were transplanted along with 4(10)^5^ competitor cells into 1 to 3 young lethally irradiated  recipients per donor. Four different donors were used per group. White blood cell chimerism was verified at 1, 2 and 5 months after transplantation by FACS analysis. Data is shown as percentage of donor derived chimerism ± SEM (**E**) Representative pictures displaying the large intestine atrophy in telomere dysfunctional mice wildtype and heterozygous for *Sod2*. (**F**) Bar graph depicting the average number of intestinal crypts per visual field at a magnification of  40X of whole mounts from n=8 (G3 *mTerc^-/-^, Sod2^+/-^*), n=7 (G3* mTerc^-/-^*, *Sod2^+/+^*); n=4   (*mTerc*^+^*, Sod2^+/-^*) and n=4 (*mTerc*^+^*, Sod2^+/+^*) mice per group.

Previous studies have shown that telomere dysfunction impairs the maintenance of high turnover organs in aging *mTerc^-/-^* mice, specifically affecting the hematopoietic system and the intestinal epithelium [38-40]. In agreement with these studies, 12-15 month old *G3 mTerc^-/-^* mice compared to age-matched *mTerc^+^*  mice exhibited anemia (Figure 3A), a reduction in bone marrow derived B-lymphopoiesis (Figure 3B, Suppl. Figure 3A-C), a reduction in thymic T-lymphopoiesis (Suppl. Figure 3D, E), and an impaired maintenance and function of hematopoietic stem cells (HSCs) (Figure 3C, D). Heterozygous deletion of *Sod2* accentuated the decrease in mature B cells in aging G3 *mTerc*^-/-^ mice (Figure 3B) but otherwise did not show consistent effects on hematopoietic parameters in *mTerc^+^* and G3 *mTerc*^-/-^ mice. As shown in previous studies [38, 40, 45], aging telomere dysfunctional mice developed a severe atrophy of intestinal epithelia compared to age-matched *mTerc^+^* mice (Figure 3E, F). *Sod2* heterozygosity did not increase the severity of crypt atrophy in aged telomere dysfunctional mice (Figure 3E, F).

**Figure 4.**
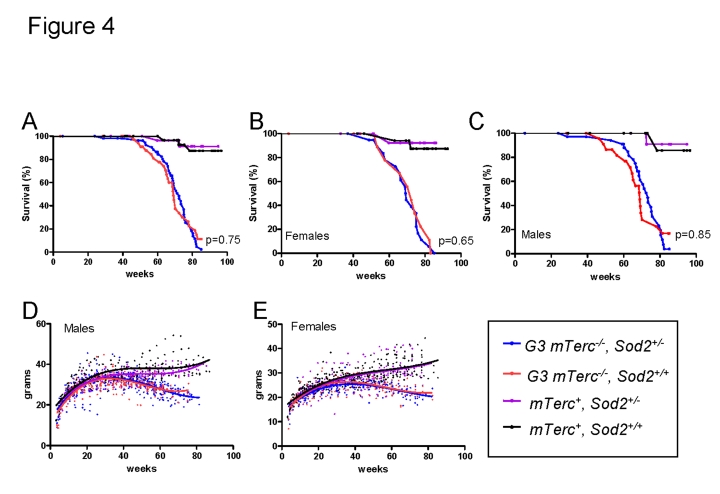
(**A**) Kaplan Meyer survival curves for G3 *mTerc^-/-^, Sod2^+/-^* (n=58); G3* mTerc^-/-^*, *Sod2^+/+^* (n=38); *mTerc^-/-^, Sod2^+/-^ (*n=31) and *mTerc^+^, Sod2^+/+^* (n=34). (**B**) Survival curves for females G3 *mTerc^-/-^, Sod2^+/-^* (n=22); G3* mTerc^-/-^*, *Sod2^+/+^* (n=14); *mTerc^-/-^, Sod2^+/-^ (*n=16) and *mTerc^+^, Sod2^+/+^* (n=19). (**C**) Survival curves for males G3 *mTerc^-/-^, Sod2^+/-^* (n=36); G3* mTerc^-/-^*, *Sod2^+/+^* (n=24);  *mTerc^-/-^, Sod2^+/-^ (*n=15) and *mTerc^+^, Sod2^+/+^* (n=15). Dot blots showing body weight of  male (**D**) and female (**E**) mice throughout lifespan in the aging cohorts.  Third order polynomial regression is shown as trendline. All mice were weighed monthly until death.

In line with previous results, an impairment in organ maintenance was associated with a shortened lifespan of telomere dysfunctional mice compared to *mTerc^+^* mice (Figure 4 A-C) correlating with an age-dependent decline in body weight (Figure 4 D, E). Heterozygous deletion of *Sod2 *did not alter weight curves (Figure 4D, E) or survival (Figure 4 A-C) of telomere dysfunctional mice. Specifically, no survival difference was observed between G3 *mTerc^-/-^Sod*2^+/-^ mice compared to G3 *mTerc^-/-^*, *Sod2*^+/+^ mice (median lifespan 72.3 and 69.1 weeks respectively, p=0.75 Figure 4A). *Sod2* heterozygosity did also not affect the incidence of spontaneous cancer in aging G3 *mTerc^-/-^* mice and *mTerc^+^* mice during the observation period of 20 months (data not shown).

## Discussion

The current study shows that SOD2 reduction does not affect stem cell function, organ maintenance, and lifespan of telomere dysfunctional mice. These results contrast with studies on mouse models of diseases, where *Sod2 *hemizygosity exacerbated disease phenotypes as (i) increasing the formation of neurotoxic plaques and tangles in APP and Tg2576 transgenic [57, 58], (ii) reducing the lifespan of G93A transgenic mice - a model for amytrophic lateral sclerosis [59], (iii) increasing diabetic neuropathy in db/db mouse [60], and (iv) increasing endothelial dysfunction in atherosclerosis prone ApoE deficient mice [61]. Together, these findings suggest that in contrast to disease conditions, telomere dysfunction *per se* does not cooperate with a decrease in anti-oxidant defense to impair organ maintenance and lifespan.

Our experiments show that heterozygous deletion of *Sod2* results in diminished SOD2 protein levels and impaired anti-oxidant defense in different organ systems of telomere dysfunctional mice including muscle and hematopoietic cells. However, this deficiency does not lead to an impairment in mitochondrial function in aging telomere dysfunctional mice, whereas it reduces mitochondrial function in aged *mTerc^+^* mice. Previous studies have shown that mitochondrial dysfunction contributes to induction of senescence in fibroblast cultures [26, 62]. The maintenance of mitochondrial function in *G3mTerc^-/-^*, *Sod2^+/-^* mice suggests that telomere dysfunction might induce adaptive responses that protect mitochondria from oxidative damage possibly involving activation of repair responses. Alternatively, telomere dysfunction may cooperate with mitochondria dysfunction *in vivo* to induce clearance of dysfunctional cells, thus maintaining a cellular pool with functional mitochondria.

There is an ongoing debate whether mechanisms that increase oxidative stress can contribute to an accumulation of nuclear DNA damage and aging. Studies with cultured human cells suggested that the contribution of mitochondrial-derived ROS to the generation of oxidative modifications in nuclear DNA is small [63]. However, *in vivo* studies with *Sod2* mutant mice have shown that *Sod2* haploinsufficiency can increase the level of oxidative modification to nuclear DNA and increase the cancer risk in very old (26 month old mice) [13]. In addition, there is evidence that impairment of DNA repair systems results in elevated ROS-mediated nuclear DNA damage, cellular senescence, and cancer formation [33, 64, 65].

In the current study, the maximal lifespan of telomere dysfunctional (G3 *mTerc^-/-^*) mice was limited to 19 months. During this observation period, *Sod2* haploinsufficiency did not accelerate the accumulation of oxidative DNA base damage or DNA double strand breaks in both telomere dysfunctional mice and *mTerc^+^* mice. These data indicate that heterozyogous deletion of *Sod2* does not cooperate with telomere dysfunction to accelerate the accumulation of nuclear DNA damage. The results from previous studies suggest that telomere independent factors may cooperate with *Sod2* haploinsufficiency in long lived wild type mice to increase nuclear damage. It appears to be surprising that nuclear DNA damage was not increased in G3 *mTerc^-/-^*, *Sod2^+/-^*mice, although the mice showed increased intracellular ROS in muscle and impaired anti-oxidant defense in response to stress induced ROS. Possible explanations include that (i) removal of oxidative DNA lesion in repair proficient mice is sufficiently efficient to counteract effects of moderately increased ROS or (ii) diffusion of mitochondria derived ROS to the nucleus is limited irrespective of *Sod2* gene status.  In agreement with the data on unchanged rates of nuclear DNA damage, the current study did not detect a cancer promoting effect of *Sod2* deficiency in telomere dysfunctional mice and *mTerc^+^* mice during the observation period of 19 month.

Together, the current study provides the first experimental evidence that an impairment of SOD2-dependent anti-oxidant defense does not cooperate with telomere dysfunction to aggravate organismal aging.

## Materials and methods

Mouse crosses and survival. *Sod2*^+/-^ mice [7] acquired from Jackson laboratories (stock number 002973) was crossed with *mTerc*^-/-^mice  for 3 generations in order to create the following experimental cohorts G3 *mTerc^-/-^, Sod2^+/-^* (n=58); G3* mTerc^-/-^*, *Sod2^+/+^* (n=38); *mTerc^+^, Sod2^+/-^ (*n=31) and *mTerc^+^, Sod2^+/+^* (n=34). Mice were kept in a pathogen free environment where they had free access to food and water.

Mice were sacrificed by CO2 asphyxiation when presented deteriorated health condition or loss of 30% of body weight. Organs were quickly removed and either frozen down in dry ice or fixed in 4% paraformaldehyde (PFA) for paraffin embedding.

Whole mounts of colon were prepared as previously described [66].

FACS analysis. FACS analysis was performed on freshly isolated bone marrow cells that were stained for 15 min on ice with the appropriate antibody cocktail. Cells were analyzed using an LSRII or FACS Calibur machine.

Bone marrow competitive transplantation. One to three young C57/BL6 mice per donor were retroorbitally transplanted after lethal irradiation (12Gy) with 8x10^5^ donor (Ly5.2) and 4x10^5^ competitor (Ly5.1) bone marrow cells. Four 15-months-old male mice per genotype were used as donors and four 12-month old Ly5.1 female mice were used as competitors in the experiment.

Chimerism was checked at one, 2 and 5 months after transplantation in white blood cells collected from retroorbital bleeding.

γ-H2AX staining in intestine sections. Three um paraffin sections were stained with primary anti γ-H2AX (Millipore 06-636) overnight in PBS, washed three times and incubated for 30 min with secondary anti mouse IgG labeld with Cy7. Slides were kept at 4°C until analysis.

Oxidative modifications to DNA. Quantification of the basal levels of oxidative purine modifications in bone marrow cells isolated from the various mouse strains was carried out by the alkaline elution assay originally described by [49] with modifications reported previously [50, 67]. Fpg-sensitive sites detection was performed in 1x10^6^ bone marrow cells per mouse as previously described [68].

ROS measurement in bone marrow cells. *Mitosox staining: *Freshly isolated bone marrow cells were loaded in staining media with Mitosox (Molecular probes. Cat. No. M36008)  5uM final concentration and incubated for 30 min at 37°C. The cells were washed once with PBS and antimycinA (Sigma Cat No. 8674) was added for a final concentration of 20uM. The cells were incubated 10 minutes, filtered and immediately analysed by FACS.

*DCFDA staining: *Freshly isolated bone marrow cells were stained for LSK (except for CD34) loaded with DCFDA 0.5 uM (Molecular probes Cat. No. C6827) and incubated for 5 min at 37°C. The cells were washed once with PBS and  antimycinA (Sigma Cat No. 8674) was added for a final concentration of 50uM. The cells were incubated 10 minutes, filtered and immediately analyzed by FACS.

ROS measurement in muscle bundles. Muscle fibers were incubated  with DHE at a final concentration of 40 μM in PBS for 30 min at 37°C. After staining, the tissue was washed in PBS and fixed using 2.2 % formaldehyde in 0.1 M Sorensen phosphate buffer (pH 7.1). Confocal images were collected with 40x objective.

Protein analysis. Whole cell extracts were prepared in RIPA buffer with cocktail of protease inhibitors and reducing agents (NaVO3 1mM, DTT 1mM, PMSF 1mM, proteinase cocktail inhibitor ROCHE Cat. No. 11836153001). SOD2 levels were determined using AntiSOD2 antibody (Santa Cruz, SC-30080) and actin levels with antiActin (Santa Cruz SC-1615).

High-resolution respirometry. Mitochondrial respiration was performed in intact bone marrow cells  and permeabilized muscle bundles as described [62, 69].

*Muscle bundles:* Respirometry of saponin-permeabilized muscle fibers was performed with the Oxygraph-2k (OROBOROS instruments) using between 10 and 25 mg of biopsy material. Measurements were performed at 37°C in the range of 200-400 μM oxygen, to avoid oxygen limitation. The experiments were performed in MiRO5 buffer (110 mM sucrose, 60 mM potassium lactobionate, 0.5 mM EGTA, 1 g/l  BSA fat free, 3 mM MgCl_2_, 20 mM taurine, 10 mM KH_2_PO_4_,  20 mM HEPES, pH 7.1).

Defined respiratory states were obtained by a multiple substrate-inhibitor titration regime: malate 2mM, octanoylcarnitine 1 mM, ADP 5 mM, glutamate 10 mM, succinate 10 mM, cytochrome *c* 10 μM, FCCP (stepwise, increments of 0.25 μM up to final concentration of maximally 1.25 μM), rotenone 0.5 μM, and antimycin A 2.5 μM. Cytochrome *c* was added to verify the intactness of the outer mitochondrial membrane after saponin permeabilization.  No visible stimulatory effect of cyt. *c* was observed in our conditions. If necessary, re-oxygenations were performed with pure oxygen.

*Bone marrow cells:* Approximately 7x10^6^ freshly isolated bone marrow cells were resuspended in 3 ml of Iscoves Modified Dulbecco's Medium (Gibco) and applied for high-resolution respirometry as above. The experimental regime started with routine respiration (defined as endogenous respiration without additional substrates or effectors). After observing steady-state respiratory flux, the ATP synthase inhibitor oligomycin (1 μg/ml) was added, followed by uncoupling of oxidative phosphorylation by stepwise titration of FCCP (carbonyl cyanide *p*-trifluoromethoxyphenylhydrazone) up to optimum concentrations in the range of 2.5-4 μM. Finally, respiration was inhibited by complex I and compex III inhibitors rotenone (0,5 μM) and antimycin A (2,5 μM) respectively.

The mitochondrial respiration data were normalized to the mitochondrial mass marker enzyme Citrate Synthase (CS) activity spectrophotometrically determined [70].

Quantitative Fluorescence In Situ Hybridization (qFISH). qFISH analysis was performed as previously described [71] in 5 uM liver and small intestine sections.

Software and analysis of data. FACS results were analyzed using FlowJo 7.2.2. Statistical analysis of the results was performed using Excel 2003 and GraphpadPrism 5.0 and image analysis with ImageJ 1.39. Chemicapt 5000 ver 15.01 was used for acquisition of images from gels and western blots. Telomere fluorescence intensity was analyzed using the TFL-Telo software from Peter Lansdorp

## Supplementary figures

Supplementary Figure 1 (**A**) Mating scheme to generate the double mutant G3 mTerc-/-, Sod2+/-.

Supplementary Figure 2 (**A**) Mitochondrial respiration of bone marrow cells. 107 bone marrow cells were analyzed by high resolution respirometry of n=5 to 7 mice per group. Results show normalized respiration of one million cells to citrate synthase activity ± SEM.  (**B**) Telomere length analysis by qFISH in  small intestine sections of n= 4 to 5 mice per group aged 12 to 18 months old. n=177 (G3 mTerc-/-, Sod2+/-), n=192 (G3 mTerc-/-, Sod2+/+); n=151   (Sod2+/-) and n=167 (Sod2+/+) nuclei were analyzed for telomere fluorescence intensity (TFI). The black line indicates the mean TFI value of each genotype and the dotted line the threshold of critically short telomeres (TFI<4000). Aging and DNA damage markers EF1-α (**C**), CRAMP (**D**) and chitinase (**E**) were quantified  by ELISA in plasma of old age matched G3 mTerc-/-, Sod2+/- (n=16); G3 mTerc-/-, Sod2+/+ (n=14); mTerc-/-, Sod2+/- (n=8) and mTerc+, Sod2+/+ (n=10) and young WT (yWT) mice (n=5). Values are arbitrary units ± SEM.

Supplementary Figure 3 Bone marrow of 12 to 18 month old mice was evaluated for: (**A**) Percentage of PreB cells defined as IgD- IgM- B220+ CD43- cells in total bone marrow cells. (**B**) Percentage of ProB cells defined as CD19+ B220+ LinB- AA4.1+  cells in total bone marrow cells. (**C**) Percentage of PreproB cells  defined as CD19- B220+ LinB- AA4.1+ cells in total bone marrow cells. (**D**) Representative FACS blot showing the reduction of thymic T-lymphopoiesis and thymic atrophy in aged telomere dysfunctional mice. (**E**) Bar graphs showing the number of thymocytes  ± SEM in n= 5 mice per group aged 12-15 months old.
