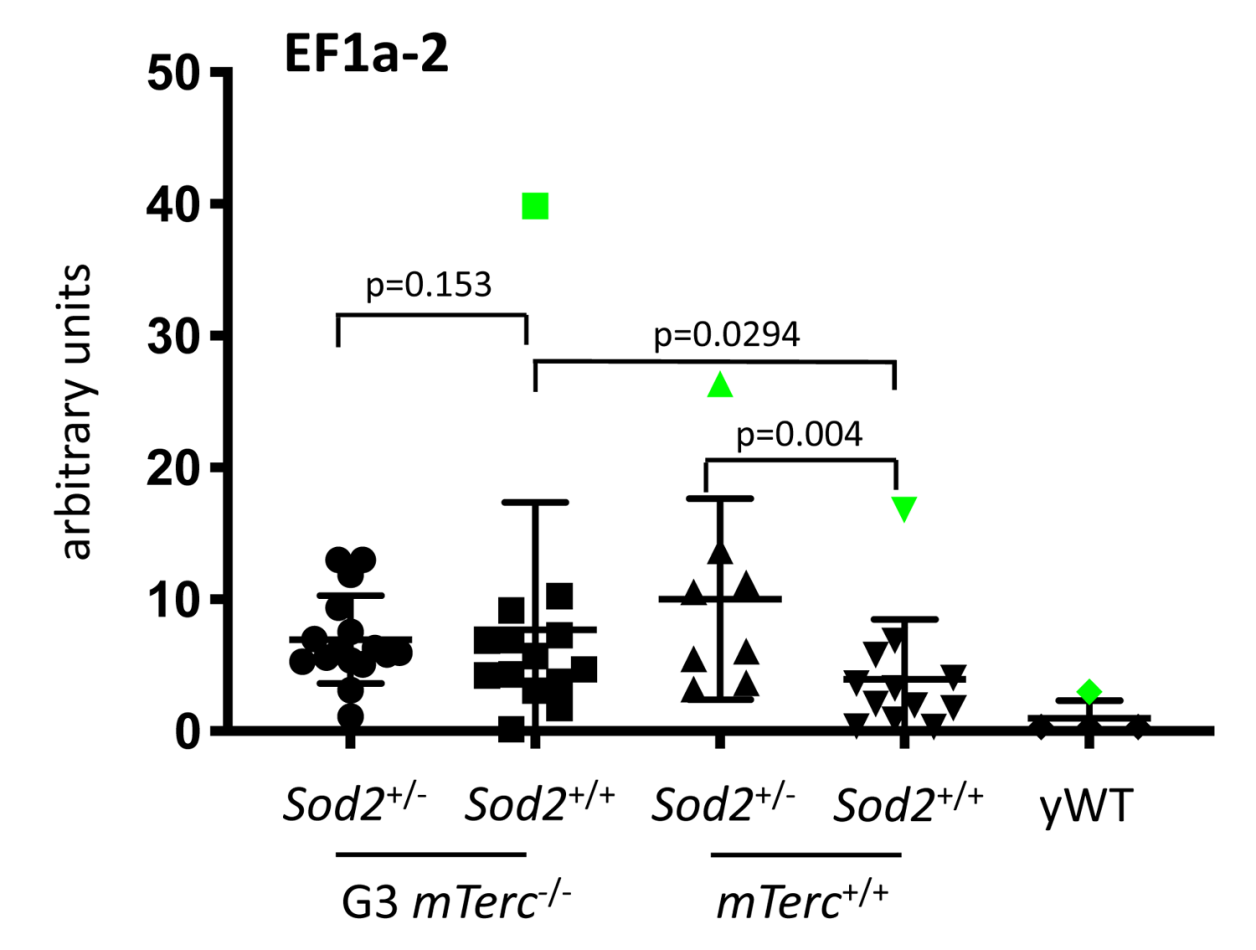# Correction for: *Sod2* haploinsufficiency does not accelerate aging of telomere dysfunctional mice

**DOI:** 10.18632/aging.102602

**Published:** 2019-12-06

**Authors:** Luis Miguel Guachalla, Zhenyu Ju, Rafal Koziel, Guido von Figura, Zhangfa Song, Markus Fusser, Bernd Epe, Pidder Jansen-Dürr, K. Lenhard Rudolph

**Affiliations:** 1 Institute of Molecular Medicine and Max-Planck-Research-Group on Stem Cell Aging, University of Ulm, Ulm 89081, Germany; 2 International MD/PhD Program, Medical School Hannover, Hannover, Germany; 3 Institute for Biomedical Aging Research, Austrian Academy of Sciences, Innsbruck A-6020, Austria; 4 Department of Internal Medicine I, University of Ulm, Ulm, Germany; 5 Institute of Laboratory Animal Sciences and Max-Planck-Partner Group on Stem Cell Aging, Chinese Academy of Medical Sciences, Beijing, China; 6 Institute of Pharmacy, University of Mainz, Mainz D-55099, Germany

**Keywords:** correction

Original article: Aging. 2009; 1:303–315. . https://doi.org/10.18632/aging.100030

**This article has been corrected: **The authors requested to replace Supplementary Figure 2C. In Supplementary Figure 2C-E the errors occurred in p-value calculation and n-number depiction. Supplementary Figure 2C contained 4 outliers (Grubb’s test) but only 1 was removed for p-value calculation. The corrected Supplementary Figure 2C is shown below and indicates the outliers in green as well as the corrected p-values. In Supplementary Figure 2E the p-value for the comparison of Sod2+/+, G3mTerc-/- mice versus Sod2+/+, G3mTerc-/-mice should read p=0.12 instead of p=0.77. N-numbers for Supplementary Figure 2C-E were 16-17 for Sod2+/+, G3mTerc-/- mice, 14-16 for Sod2+/+, G3mTerc-/-mice, 7-8 for Sod2+/+, mTerc+/+ mice, 12-14 for Sod2+/+, mTerc+/+ mice, and 4-5 for young wildtype (yWT) mice. Aging markers were determined as previously described (Reference 16 of the manuscript). Statistical analysis was performed using the unpaired t-test with GraphPad Prism software. These corrections do not change any of the conclusions of the publication. The corrected Supplementary Figure 2C is provided below.

**Supplementary Figure 2C:**