# Supplementary figures and images for: Sod2 haploinsufficiency does not accelerate aging of telomere dysfunctional mice
Source: Aging (Albany NY). 2009 Mar 5;1(3):303–15. doi: 10.18632/aging.100030 (PMC2830048; doi:10.18632/aging.100030)

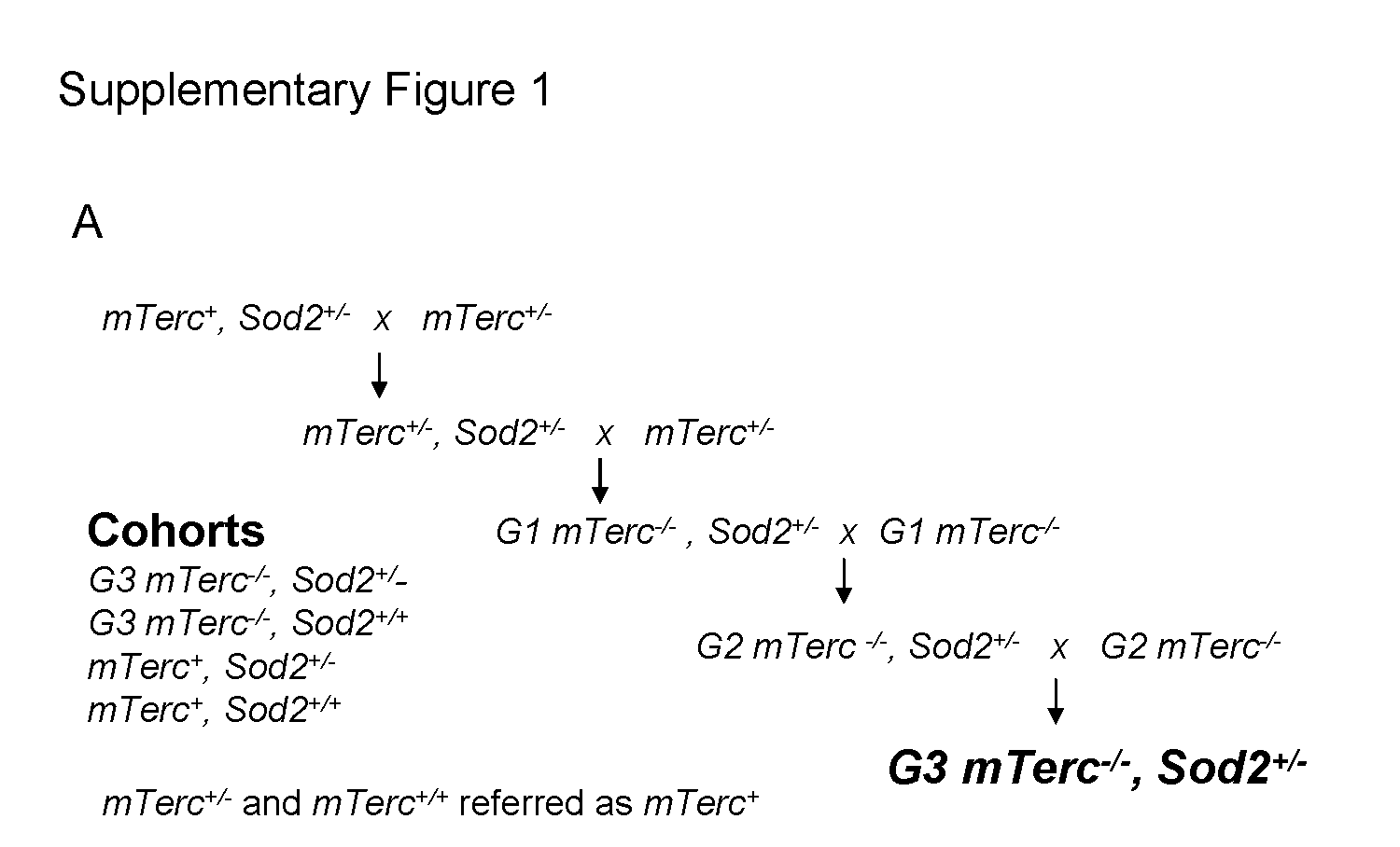

Supplement: Supplementary Figure 1 — (A) Mating scheme to generate the double mutant G3 mTerc-/-, Sod2+/-. [file aging-01-303-s001.tif]

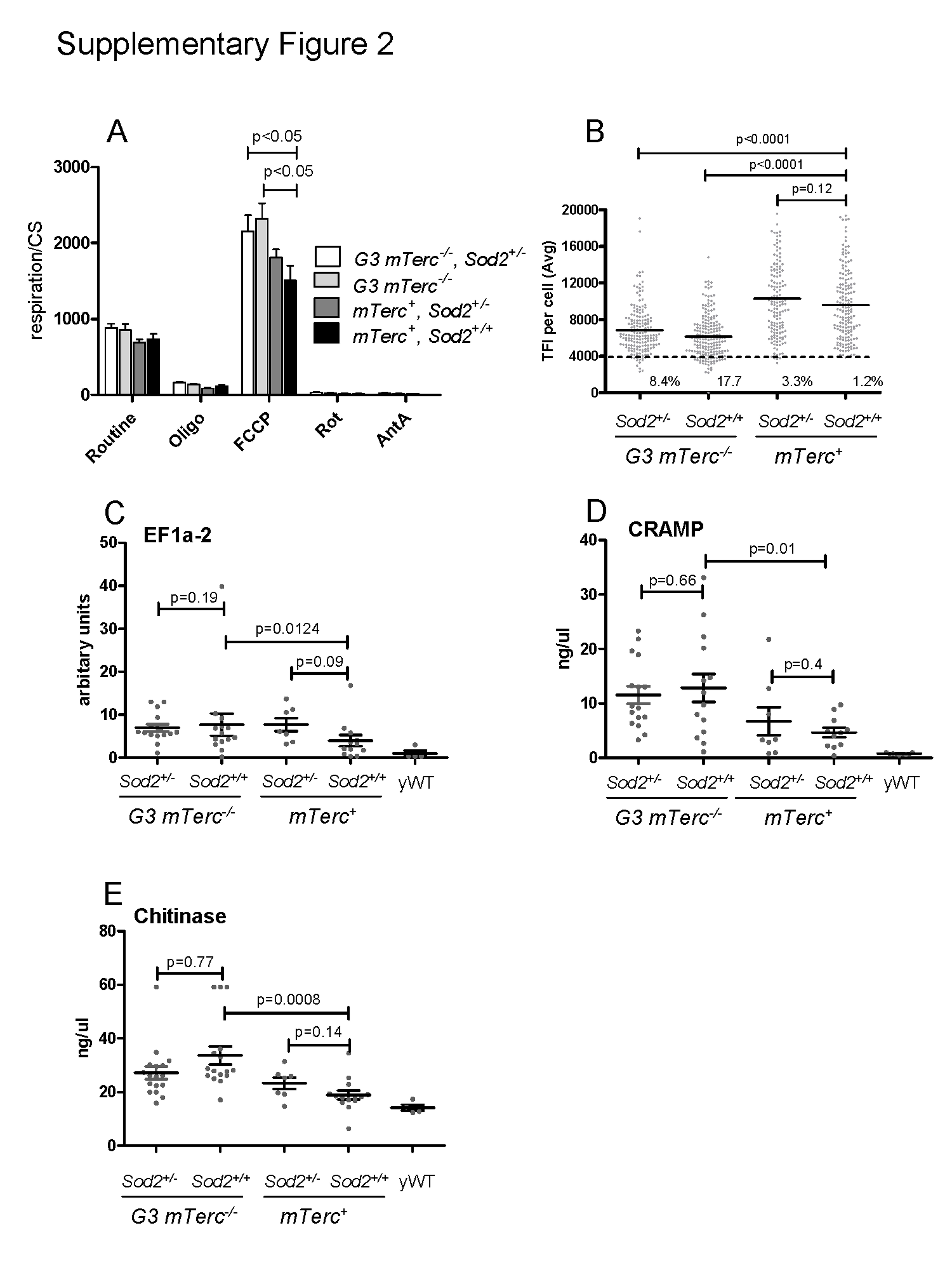

Supplement: Supplementary Figure 2 — (A) Mitochondrial respiration of bone marrow cells. 107 bone marrow cells were analyzed by high resolution respirometry of n=5 to 7 mice per group. Results show normalized respiration of one million cells to citrate synthase activity ± SEM. (B) Telomere length analysis by qFISH in small intestine sections of n= 4 to 5 mice per group aged 12 to 18 months old. n=177 (G3 mTerc-/-, Sod2+/-), n=192 (G3 mTerc-/-, Sod2+/+); n=151 (Sod2+/-) and n=167 (Sod2+/+) nuclei were analyzed for telomere fluorescence intensity (TFI). The black line indicates the mean TFI value of each genotype and the dotted line the threshold of critically short telomeres (TFI<4000). Aging and DNA damage markers EF1-α (C), CRAMP (D) and chitinase (E) were quantified by ELISA in plasma of old age matched G3 mTerc-/-, Sod2+/- (n=16); G3 mTerc-/-, Sod2+/+ (n=14); mTerc-/-, Sod2+/- (n=8) and mTerc+, Sod2+/+ (n=10) and young WT (yWT) mice (n=5). Values are arbitrary units ± SEM. [file aging-01-303-s002.tif]

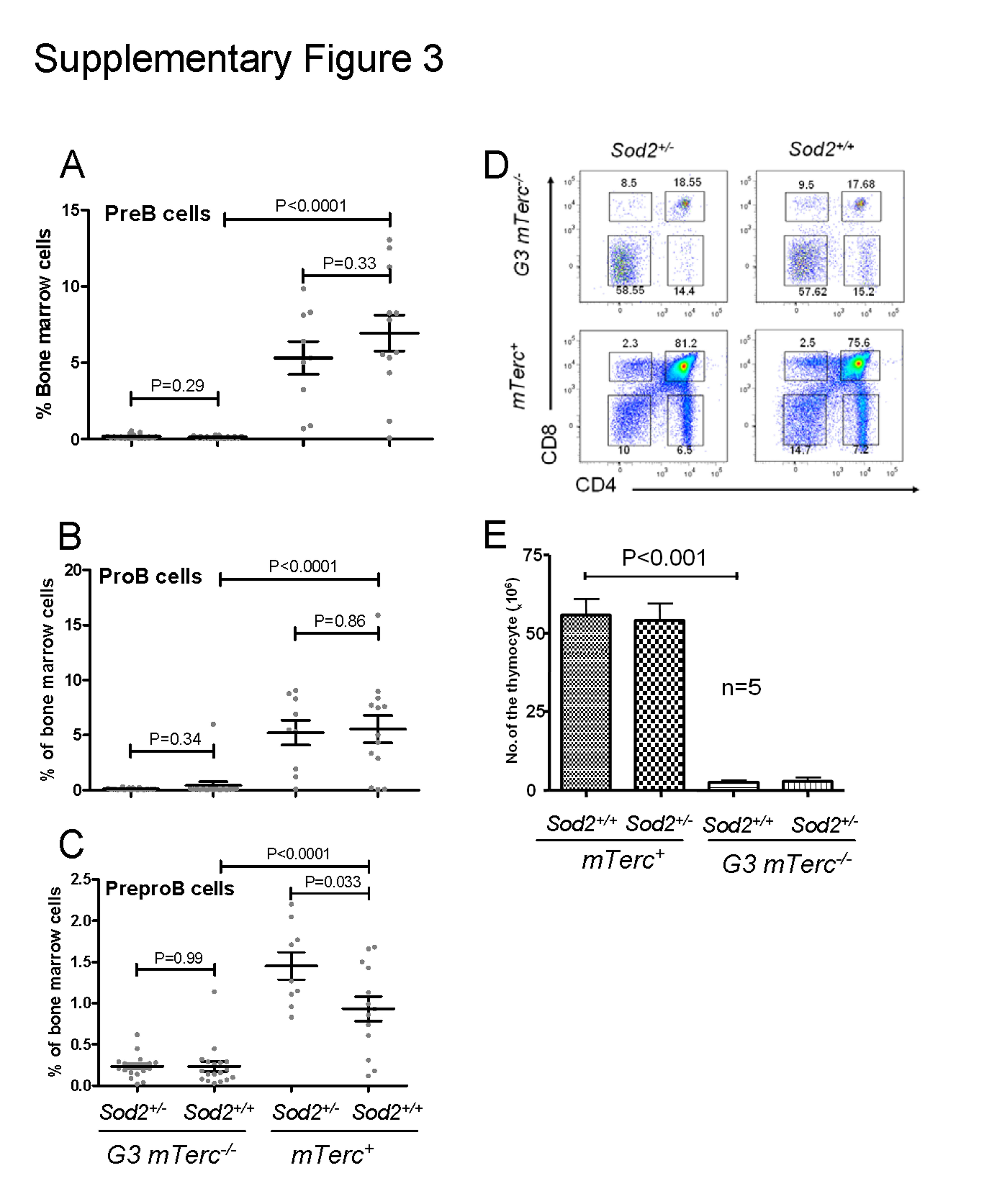

Supplement: Supplementary Figure 3 — Bone marrow of 12 to 18 month old mice was evaluated for: (A) Percentage of PreB cells defined as IgD- IgM- B220+ CD43- cells in total bone marrow cells. (B) Percentage of ProB cells defined as CD19+ B220+ LinB- AA4.1+ cells in total bone marrow cells. (C) Percentage of PreproB cells defined as CD19- B220+ LinB- AA4.1+ cells in total bone marrow cells. (D) Representative FACS blot showing the reduction of thymic T-lymphopoiesis and thymic atrophy in aged telomere dysfunctional mice. (E) Bar graphs showing the number of thymocytes ± SEM in n= 5 mice per group aged 12-15 months old. [file aging-01-303-s003.tif]
